# Sketches of the Medical Schools of Paris; Including Remarks on the Hospital Practice, Lectures, Anatomical Schools, and Museums; and Exhibiting the Actual State of Medical Instruction in the French Metropolis

**Published:** 1816-02

**Authors:** 


*Sketches of the Medical Schools of Paris; including Remarks on the Hospital Practice, Lectures, Anatomical Schools, and Museums; and exhibiting the actual State of Medical Instruction in the French Metropolis.* By JOHN CROSS, Member of the College of Surgeons in London, Corresponding Member of the Société Médicale d'Emulation at Paris, and late Demonstrator of Anatomy in the University of Dublin. 8vo. pp. 208. London, 1815. With two Engravings.

MR. Cross passed the winter of 1814 in Paris; and the present publication is dictated by the laudable desire of communicating to his countrymen all the information which, during his short residence, he was enabled to glean respecting the medical institutions of that "extraordinary metropolis." The remarks in which he occasionally indulges, bespeaks, we think, on the whole, rather good sense and impartiality, than profound and comprehensive views of the subject upon which he writes. His work is sketched in an easy and interesting style; but is defective in that which constitutes a peculiar excellence of scientific disquisitions—precise and luminous arrangement. Objects which require to be minutely examined are best seen in an isolated form. Irregularity may delight the imagination, or give enchantment to the productions of the poet: but order is the luminary of science; and confusion and obscurity invariably result, whenever it ceases to shed a benignant influence on the investigations of the philosopher.

In analysing the publication of Mr. Cross, we shall attempt a route somewhat different from that which he has himself pursued, and one better calculated, in our opinion, to exhibit clear and distinct views of the various objects which the British reader will feel an interest in contemplating. We shall first describe the French system of medical education, the schools and methods of instruction as they regard the different classes of medical practitioners, with every thing which relates to the œconomy and regulations of the metropolitan hospitals; and, secondly, trace, under separate heads, those peculiarities of practice which form at present the distinguishing features of the French schools, and expose, as clearly as we are able, the actual state of medicine, or rather surgery, in that enlightened but unhappy country; for to the exposition of surgical education and practice are the remarks of Mr. Cross almost exclusively restricted.

Before, however, we proceed, it may not be amiss to present a hasty sketch of the various classes into which the great body of medical practitioners in France is distinguished; the course of instruction which they are respectively required to follow; and the situations and offices they are destined to fill in the field of civil practice. The education of medical officers for the army and navy will be cursorily explained in our account of the Parisian schools.

The private practitioners of medicine in France may be divided into three great classes.

1. *The Doctors of Medicine and Surgery;* who can only graduate at Paris, Strasbourg, or Montpellier,* after a course of study of more than four years, conducted upon a plan which we shall presently specify. No one can legally practise as physician or surgeon, without taking a degree under these regulations. They are forbidden to dispense medicines; and, therefore, chiefly practise in cities or the larger towns. Though directed by law to restrict his exertions to that branch of the healing art for which he may have graduated, the doctor of medicine or of surgery often practises both branches indiscriminately.

2. *The Officiers de Santé;* men of more limited education, who reside principally in small towns and villages. They are not required to study at the regular schools, but may obtain a licence to practise without going out of their respective districts, after having lived six years with a regular doctor, or five in a country hospital, and subsequently undergone an examination by a jury of medical men, appointed in every department of France for that purpose. They can only practise in the district wherein they have been examined, and under certain restrictions. They can only perform the great surgical operations under the eye of a doctor, or in urgent cases. Medicines for their own patients they are allowed to supply, but forbidden to keep shops for the sale of drugs. Thus the French *Officier de Santé* resembles, in the great outline, our sur-

* These three schools were established in 1795, under the title of *l'Ecole de Santé.* In 1803, they were made colleges for granting medical and surgical degrees; and, in the same year, two similar institutions were formed at Turin and Mayence.

geon-apothecary; but in education and in rank he is decidedly inferior to the general practitioner of Britain.

3. *The Pharmaciens*, or druggist-apothecaries; whose office it is to sell drugs and dispense the prescriptions of the regular doctors. There are schools of pharmacy, established expressly for their education at Paris, Strasbourg, and Montpellier.* The juries in the departments, by whom the *Officiers de Santé* are examined, possess, in common with these three schools, the privilege of appointing country *pharmaciens*. They cannot be admitted to examination, or receive licences to act, until they have gone through eight years of servitude as apothecaries, and attained their twenty-fifth year.

The practice of midwifery is almost exclusively in the hands of women; and we shall presently have to notice an admirable institution for the education of female students in this important, but with us too much neglected, branch of the healing art.

We now proceed directly to our subject. In the third year of the French republic, an *Ecole de Santé* was established at Paris, by a decree of the National Convention. The object of it was to provide the hospitals, and particularly the military and naval establishments, with regularly educated medical officers. The noble edifice of the late Academy of Surgery was appropriated to this purpose.

Previously to this period, nothing like a system prevailed in the practice of medicine and surgery. The profession was even in a more degraded state than during the old order of things before the Revolution, when there were almost as many universities as provinces; and the *summi honores* of medicine were indiscriminately conferred upon all who had the inclination or ability to *pay* for them.

Twelve professors, twelve assistant-professors, a director, inspector, demonstrator-in-chief, librarian, conservator of the Museum, draughtsman, and modeller, were appointed by the Convention to *L'Ecole de Santé;* and a hospital *(l'Hospice de Perfectionnement)* and dissecting-rooms, of which we shall hereafter speak, at the same time instituted. During the six winter months, lectures were to be delivered on anatomy and physiology; medical chemistry and pharmacy; operative surgery; and doctrine of Hippocrates, and history of rare cases. During the summer, medical natural history; doctrine of health; internal (medical)

---

* And subsequently at Turin and Mayence.

pathology; external (surgical) pathology; medical jurisprudence and history of medicine; midwifery.* Clinical lectures were also delivered, during the whole year, at *L'Hospice de Perfectionnement,* at *L'Hotel Dieu,* and *La Charité.*

The *Eleves de la Patrie,* or national students, were young men, between the age of seventeen and twenty-six, sent from every part of France to receive gratuitous medical instruction at *L'Ecole de Santé* of Paris, Strasbourg, or Montpellier. The number to be received at the Parisian school was limited to three hundred. They were divided, according to their advancement, into three classes; and for each an appropriate plan of theoretical and practical instruction was marked out.

These national students were eventually destined, as we have before observed, to fill the situations of medical officers in the French hospitals and army; and, in order to secure their regular attendance at the schools, "each professor was directed to call over their names twice in ten days, and those who were found absent three times out of ten, were referred to the committee for regulating public instruction, and reprimanded or dismissed."

"To ascertain the progress of the national students at the conclusion of each course, those amongst them who had followed it were assembled, and three questions proposed, to which they gave a written answer at the end of an hour and a half; from which the professors judged of their advancement. Besides this, there was held annually a general examination of all the students, when premiums were given to the most worthy, and those who did not satisfy the professors were discharged, to make room for others." p. 7.

That some such regulations as these are wanted in the British schools of medicine it is almost unnecessary to add.

Such was the plan of medical education in Paris towards the close of the last and commencement of the present century. It now remains for us to exhibit the actual state of instruction in that capital, as observed by Mr. Cross; and it will be obviously proper to consider, first, in one view, the regular school of medicine, its immediate dependencies, and the hospitals connected with it. We shall afterwards pass on to the detached and minor institutions.

---

* One course of four months for the national students; and one of two months for female practitioners.

*L'Ecole de Medecine*, formerly *L'Ecole de Santé*, is the universal school of public instruction in Paris, and the only institution there for granting medical and surgical degrees. It is supported by Government; and the professors receive, each, an annual stipend of five thousand *francs* (upwards of 200*l*). The following lectures were delivered during the last winter season, from November to April: — Anatomy and physiology, every day at ten o'clock; medical chemistry and pharmacy, three days in the week, at twelve; operative surgery, on the three intervening days, at the same hour; demonstration of surgical instruments, every Thursday, at one. All the lectures are public and accessible without ceremony or expence. Those who attend them may be arranged in three classes.

First. The individuals who frequent them from mere curiosity, or thirst for knowledge. This class is the least numerous.

Secondly. Those who are studying for a medical or surgical degree. The term of study is about four years, and the plan of education for physician and surgeon precisely the same. Each of them must present himself at the commencement of every three months, to take a fresh *inscription*, or ticket, for the lectures; but regular attendance at the lectures or hospitals is voluntary. No one can be inscribed for more than one course of lectures at a time; and the expence of each *inscription* is from twenty-five to thirty shillings. This class of students, including generally the more respectable, has the privilege, not granted to the former, of asking for a degree at the end of four years. The examinations to which the candidate for a degree must submit are five, all public, and two of them in the Latin language. Anatomy and physiology form the subject of the first; pathology and nosology, of the second; materia medica, pharmacy and chemistry, of the third; doctrine of health and medical jurisprudence, of the fourth; whether the object of the candidate be a medical or surgical degree. The fifth examination for the former is on practical medicine; for the latter, on practical surgery. A thesis on some medical or surgical subject is, moreover, to be written by the candidate, and defended in the public theatre. The expences of graduation are, in either case, the same; about twenty-five pounds. The income arising from this source, and from the *inscriptions*, is divided among the professors.

Thirdly. The *Eleves de l'Ecole Pratique* (students of the practical school). This class will be best understood after reading the account, given by Mr. Cross, of *L'Ecole Pratique*. In this are included " the professors of *L'Ecole de Medecine*, the surgeons and physicians of hospitals, demonstrator *en chef*, *prosecteurs*, and assistants of the anatomical school, and what were formerly called national students, who are practically employed as assistants to the professors in the dissecting-rooms or in the hospitals. These students of *L'Ecole Pratique* are received after an examination *au concours* by the professors; they are examined regularly, to shew their progress in their studies, and are admitted to be candidates for annual prizes, which are bestowed on the writers of the best dissertations, &c. Besides these means to insure their attention to their studies, and incite them to make great exertions, they have other advantages; they are exempted from paying for their *inscriptions* as well as for their doctor's degree in medicine or surgery, which is given to them, gratis, after the regular course of study, and the examinations which I have explained in speaking of the second class." The students of the practical school then are young men " who have little money to pursue their education, or who are intending to become medical officers in the army and navy, to which they are admitted after a longer or shorter course of study, according to the need of them, or the proofs they give of advancement in their profession, without having taken their doctor's degree." They possess, moreover, " a great privilege in being afterwards enabled to become candidates for the higher offices in the school, to none of which are those eligible who were merely common students and paid for their inscriptions." These favoured *eleves* have the preference of subjects whenever there happens to be a dearth in the dissecting-rooms. Mr. Cross complains of their being received with too little attention to their previous general education.

The mode of electing officers to *L'Ecole de Medecine*, in the event of a vacancy, cannot be sufficiently admired. Mr. Cross, in illustration of it, details the process by which a *chef des travaux anatomiques* was lately chosen. We warmly recommend a perusal of it to all those whose province it may be to preside over the election of candidates for situations of great responsibility in the public medical institutions of Britain. It will suffice here to observe, that the candidates for the office in the Parisian school were subjected to three methods of trial,* before a jury composed of seven professors of the faculty of medicine; and the subjects and nature of the examination were such, that none but an able and expert anatomist could hope to pass the ordeal. We think that there are some in this island, even among those growing grey in the business of anatomical instruction, who would feel rather queerly in submitting to such an examination.

Chaussier is the Lecturer on Anatomy at *l'Ecole de Medecine.* The preparations for lecture are made, Mr. Cross informs us, with great care; the dissections clean and elegant; the lectures tediously minute, correct in the descriptions of the isolated systems, but defective in surgical anatomy. He here saw a new mode of opening the cranium exhibited, which "was by trephining it in four places, introducing a flexible spatula through the trephined holes so as to detach the *dura mater*, and then removing the skull-cap by sawing the cranium from one hole to the other. A plan for examining the thorax or abdomen posteriorly, by removing parts of the spine, was proposed. It may be employed with great advantage when we wish to obtain views of the internal parts, whether in a sound or morbid state, with the least possible injury and derangement of them. These lectures were frequented, last winter, by more than a thousand students.

Dupuytren presides at the lectures on operative Surgery. About twelve hundred students were present when inguinal hernia was the subject of discussion. Six bodies were brought in for the purpose of demonstrating the external and internal views of the anatomy of the parts in the male and female. The external view was developed "rather according to Scarpa's account of it, and without any reference to the English authors as to fasciæ, &c." Mr. Cross

* The first trial consisted of *written* answers to the questions proposed: it was on three subjects, and the answer to be returned in a stated time. Description of the structure and physiology of the ear, within six hours; of the organ and sense of smell, four hours; survey of the duties of the situation to which they aspired, &c. five days. Second trial: Preparations, by injection, of the internal maxillary artery; of the vena azygos, subclavian veins and lymphatic trunks; and dissection of the great sympathetic nerve, fifteen days: together with public performance of the operation, by incision, for fistula lachrymalis, amputation at the shoulder-joint, and partial amputation of the foot. Third trial: *Verbal* demonstration and criticism of the anatomical preparations of the competitors, twenty minutes; and the same time for a demonstration of the nasal duct, its diseases, and the surgical operations requisite to remove them.

complains that he seldom learnt less from any good practical lesson than from this.

The *Museum of l'Ecole de Medecine,* though well supplied with skeletons, dried diseased bones, and coarse blood-vessel preparations, is very defective in the more delicate specimens of minute structure.* There are some examples of injection of the lymphatic trunks, and a few dissections of the cervical, thoracic, and abdominal nerves; but, fœtuses and monstrosities excepted, not thirty specimens of natural or morbid parts, preserved in alcohol; no moist preparation of the minute structure of the organs of sense or internal viscera; no mercurial injection of the absorbents or excretory duct of the testis. But, to compensate for the want of these, much better recent dissections for lecture are prepared than in the London schools; and our wonder at the inattention of the French to the preservation of moist anatomical pieces will cease, when we reflect upon the facility with which recent subjects for dissection may always be procured. A new instrument for injecting the lymphatic system is described, and illustrated by a wood-cut. It consists of a fine glass tube, four or five inches long, introduced into another, three times the length, of much larger calibre, and properly secured with sealing wax. The former is then bent to a right angle near the middle, by means of a candle and blow-pipe; and drawn out into a capillary tube at the extremity by a similar process. If the tube be hermetically closed at the point, a small portion of it may be broken off. The angle of the tube may be varied according to the position of the vessels which it is the object of the operator to inject. Mr. Cross considers this instrument useful in injecting the absorbents of the liver and intestines; but that it is not superior to others, when employed upon the extremities. There are rooms attached to the Museum for models in wax. Of these, Mr. Cross speaks in terms of the warmest approbation; a room also filled with ancient and modern surgical instruments, bandages, &c.; and another, appropriated to a collection of medicinal substances, for the lectures on materia medica. An extensive library is open to all medical students three days

* Mr. Cross makes the same complaint with respect to Cuvier's Museum of Comparative Anatomy. at the *Jardin des Plantes*: and that the best preparations there are ill displayed, "lying at the bottom of bottles, half filled with dirty spirit, covered over with ground glass, and closed by putty."

a week, but peculiar privileges in the use of the books are granted to the students of *l'Ecole Pratique.* The Museum is always accessible to students and strangers, and to public curiosity three days a week.

*L'Hospice de Perfectionnement* is contiguous to *l'Ecole de Medecine,* and now appropriated exclusively to the reception of the subjects of surgical operation.* The two male wards contain about twenty beds; the female, twelve. Petit Radel and Dubois are the surgeons; M. Patrix, "a sort of resident assistant surgeon." Daily clinical lectures, from seven o'clock till nine, are announced; but not formally given, by Dubois. Operations are performed three days a week, after the surgeon's visit; and, on the three intervening days, out-patients are seen in the operating theatre by Dubois; who, after the patient has publicly recited before the students the history of the case, dictates aloud his prescription to several attendant amanuenses, by whom it is written down. The room is commonly much crowded, but the greatest order and dispatch prevail. Morbid dissections are publicly made in this room, immediately after the surgeon's visit to the wards, and they are admirably conducted; the history, progress, and treatment of each case being drawn up and recited aloud by Patrix. All the business here is concluded ere the lectures at *l'Ecole de Medecine* begin. The wards of this hospital are accessible to every one; all the branches of instruction gratuitously attended, except by those studying for a degree, who must purchase *inscriptions* for the clinical lectures. Beclard, the present *chef des travaux anatomiques,* gives public and gratuitous demonstrations every day, at two o'clock, in the operating theatre of this hospital. They comprehend human anatomy and physiology; surgical operations, pathology, and the operative part of midwifery. At one of these demonstrations, witnessed by Mr. Cross, the attachments of the femoral muscles were exposed, and traced upon the skeleton, with great accuracy and minuteness; their action long and ably detailed. But the connection of anatomy with surgical operations and diseases was not noticed: the site and relations of the femoral artery very imperfectly delineated, and not a word said about the fascia This last was certainly a great omis-

* It was at first intended for the reception of extraordinary cases, and for experiments with new remedies. Formerly this edifice was the *Convent des Cordeliers,* noted as the scene of jacobinical meetings during the revolution.

sion. No one can appreciate more deeply than ourselves the importance of a correct study of the fascial system to the anatomist and surgeon; but from the warmth with which Mr. Cross expresses himself upon it, we rather suspect that he is *touched* with the rage for fasciæ, an epidemic of recent origin in certain of the London schools, and the symptoms of which we have lately had an opportunity of observing in a professional friend ; who, on being asked his object in abandoning an excellent country practice for "a winter in London," replied, "*to dissect fasciæ.*"

The *dissecting rooms* of *l'Ecole de Medecine* are situated behind *l'Hospice de Perfectionnement.* They consist of six distinct buildings, similar in size, separated by short distances; each, a single room, capable of containing about twenty dissecting tables. The *chef des travaux anatomiques* presides in this establishment. Subjects are obtained from the hospital on his mandate, or that of the Professors of *l'Ecole de Medecine.* Dissections, during summer, are prohibited by the police; and *body-snatching* is neither allowed nor necessary. Anatomical assistants are selected from among the students of *l'Ecole Pratique,* to assist and instruct the common students; to make injections; and prepare subjects for lecture. It is not absolutely required of the student for a degree to engage in these dissections; but all are permitted to dissect, when subjects are plentiful. The cost of a subject is about six or seven shillings. Dissections are seldom well performed here. The work of Boyer on Anatomy is almost exclusively employed. Mr. Cross speaks rather contemptuously of it. A word with him on this subject bye and bye ! *L'Ecole de Medecine* is destitute of a professorship of comparative anatomy.

The two other hospitals, connected with *l'Ecole de Medecine,* and principally frequented by students during the winter, are *l'Hotel Dieu* and *La Charite;* and of these we shall next speak, so as to complete our account of the regular Parisian school, ere we proceed to consider the minor and isolated institutions. The expenditure of all the civil hospitals is regulated by one central board of administration. Certain taxes and charitable donations supply a part, and the deficiency is made up by the Government. No Parisian hospital is wholly supported by voluntary contributions.

*L'Hotel Dieu* contains generally about two thousand patients. There are many more medical than surgical cases ; and eight or nine physicians are attached to it. They deliver gratuitous clinical lectures at their morning visits throughout the whole year.

The two surgeons are Pelletan and Dupuytren: one takes charge of the male; the other, of the female department; and they change annually. The offices of house-surgeon and dresser are filled by the students of *l'Ecole Pratique*, under the designation of *eleves internes* and *externes*. The *eleves externes*, or dressers, are elected annually by the physicians and surgeons, after verbal and written examinations. The appointment is gratuitous, and may be held for one or two years: their number is unlimited. Last year, more than a hundred were attached to the *Hotel Dieu*. The *eleves internes*, house-surgeons, are elected from among the *eleves externes*, of two years' standing, and solely recommended by their superior attainments. Separate apartments are allotted to them in the hospital; and each receives, exclusive of board and lodging, an annual salary of about twenty guineas. The situation may be held for two years.* They have days of successive attendance at the *Salle de Garde* to receive accidents; and, in the absence of the physicians and surgeons, the immediate care of all the patients devolves upon them. None of the practical situations for students can be purchased here, as in the London hospitals. Merit only can get access to them. The observations of Mr. Cross upon this subject are alike pointed and correct.

M. Pelletan, last year, had the male ward; it is so large as to contain about two hundred and twenty beds, and the patients are arranged according to the nature of their complaints. This zealous veteran commenced his visit every morning at seven o'clock, in severe winter, by candle-light; and the sound of a bell announced his arrival to the students. Twice or thrice a fortnight, on uncertain days, he called over a list of the *eleves externes* and *internes* attached to his ward, in order that the absentees might be reprimanded or dismissed. His visit commonly occupied about two hours, when clinical lecture began. After this, the out-patients were admitted to receive advice. Mr. Cross expresses himself much pleased with the clinical lectures of M. Pelletan.

The female wards, last year under the care of Dupuytren, contain about one hundred and twenty beds. This surgeon is less regular in his morning visits than his col-

---

* There are about twenty at l'Hotel de Dieu, and a limited number in every hospital.

league; but his attention to the feelings and comforts of his patients is most exemplary. Capital operations are frequently performed during the morning visit of the surgeons, without ceremony or bustle, and even without removal of the patient to the operating room. Mr. Cross more than once saw the operation for hernia thus quietly conducted.

Pelletan and Dupuytren now deliver the clinical lecture alternately five mornings a week during the whole year, in the operating room. They are accessible to students of every description; but *inscriptions* to them must be purchased by those intending to graduate. The *eleves internes* and *externes* attend at these lectures to render an account of the patients committed to their care, and assist the recollection of the professors respecting the particulars of the cases; for of these no written history is preserved. The wards of *l'Hotel Dieu* are open to every student at all hours, without fee, ticket, or ceremony.

Patients, affected with chronic diseases, are sent from *l'Hotel Dieu* to a small hospital, *La Pitié,* in the environs of Paris. Behind this is a large building for dissections: it consists of three large rooms, capable of containing ninety tables. Each room is provided with an articulated skeleton, and with a smaller adjoining apartment, where a more experienced student should always be, but seldom is, found, to assist the younger, and superintend their dissections. All the dead of *l'Hotel Dieu* are brought here; and, when dissected, are regularly removed for interment. This establishment, of which M. Serres, a resident physician at *l'Hotel Dieu,* is director, and who daily gives an anatomical demonstration, forms, in fact, a dissecting school for the students of *l'Ecole Pratique;* but, as there are more bodies than they can cut up, any one may dissect by paying five shillings for a subject: and the reader will share the astonishment of Mr. Cross, on learning, that the small sum thus raised suffices to defray the chief expences of this great institution. Four hundred students were employed there last winter, notwithstanding its inconvenient distance from the principal hospitals and lecture rooms. Here too, Mr. Cross expresses his regret that "little good dissection was going on."

*La Charité* is smaller than *l'Hotel Dieu.* About a hundred beds are appropriated to surgical patients. Of these, one-third is occupied by women. Deschamp and Boyer are the surgeons, but they rarely attend. Hence the care of the surgical cases devolves on M. Roux,* assistant surgeon, who performs his duties in a very zealous and exemplary manner. He visited the hospital every morning last winter soon after seven o'clock. His arrival was announced by a signal, and the names of the dressers and house-pupils appointed here as at *l'Hotel Dieu*, in a number proportioned to the size of the hospital, were called over, ere he entered on his round. Few remarks were made in the wards; and if the visit were over before nine, he engaged in conversation with the students till that hour, when clinical lecture began, unless superseded by some surgical operation, six mornings a week in the operating room. At conclusion of lecture, the out-patients were received; and M. Roux rarely left the hospital till near eleven.

The mode in which these lectures are conducted appears to us so excellent, so unlike any thing which it has been our lot to witness in the British schools, that, in justice to the indefatigable assistant-surgeon of *La Charité*, we shall transcribe the account given of them by Mr. Cross.

"M. Roux gives considerable attention to his clinical lectures, and renders them useful to all classes of students. He keeps no written history of the cases, but the name of every surgical patient admitted into the hospital is put down in a book; and furnishes, sooner or later, some remarks for the *leçon clinique.* The lectures are varied so that they are sometimes examinations; to be able to answer at which, the *eleve interne* or *externe* is obliged to attend closely to the cases under his care; and I found that many of *les eleves internes*, who are the best-informed students, kept well-written histories of all the important cases, which they might be called to give an account of at the clinical lectures. Half the lecture is, indeed, sometimes given by the student, in reciting before the whole class the history of some case, the received notions respecting the disease under which the patient labours, and its progress since admission; and the professor has little more to do than to correct the errors, and fill up the omissions made by the student, and to add such remarks as are the result of his own experience. No students who are present at the lectures, except those of *l'Ecole Pratique*, take any share in relating cases, or answering the questions proposed by the Professor. Every case is marked on the day when it is mentioned in the clinical lecture, in order that no interesting case may go unnoticed. The remarks are not con-

* This is the gentleman who was engaged the year before last in visiting the London schools. We shall lose no time in noticing his *Relation chirurgicale d'un voyage en Angleterre*, as soon as it shall have appeared. It has been some time since announced in the French Journals, and cannot fail to be a very interesting work.

fined to the history of complete cases, nor of the diseases of patients just admitted; but reports are often made, at a few days' interval, of the progress of any extraordinary or very instructive case, of the effects of any mode of treatment that had been discussed in a former lecture, and since put in practice. A patient rarely dies in the hospital, or is discharged from it, without his case being noticed in the *leçon clinique*; and no important operation is ever performed in the theatre without becoming the subject of comment and discussion. Too little value is set upon the morbid dissections; but the bodies are constantly inspected, and the diseased parts removed by operations examined in the public theatre." Page 149—151.

A private course of evening lectures is given at *La Charité* by Roux, accessible only to those who purchase tickets.

The medical wards at *La Charité* are numerous. Corvisart formerly presided over the *clinique interne*, and had a clinical ward exclusively entrusted to him, containing fifty beds, with power to fill it by the selection of interesting cases for his lectures from all the Parisian hospitals; but Corvisart no longer fills this department, and common cases occupy the select ward. Clinical lectures on internal diseases are now inattentively delivered and ill-attended: but those intending to graduate must take out *inscriptions* for them, although attendance is optional. There is gratuitous admission to the *clinique externe*, and medical and surgical practice of the hospital. The anatomical dissections, once carried on there, have been recently discontinued by an order from *l'Ecole de Médecine*.

Here our account of the regular medical and surgical school of Paris terminates; but, in order to present a connected sketch of the whole system of French medical education, we shall deviate more widely than ever from the track followed by Mr. Cross, and render a short account of two other institutions: the one, *l'Ecole de Pharmacie*, of which we have before spoken, for the education of apothecaries; the other, *l'Hospice de la Maternité*, a part of which is devoted to a regular plan of instruction for female practitioners in midwifery.

*L'Ecole de Pharmacie*, of Paris received its present form in 1803. Public lectures are delivered on botany and the natural history of medicinal substances, pharmacy, and chemistry. In addition to this slender information, we may add, that the professors of the pharmaceutic school "are charged to visit, at least once a year, the shops and warehouses of the apothecaries and druggists, in order to ascertain the good quality of the drugs and simple and compound medicinal preparations; in which visit they are accompanied by two doctors and professors of the medical school, and by a commissary of police. They are also commissioned to watch over and examine the herbalists."* Strange as it may seem, France is without a national Pharmacopœia. The Code Pharmaceutique of Parmentier, is the only work of this sort which has appeared under the sanction of a public body.

*L'Hospice de la Maternité* has risen, since the revolution, upon the ruins of the old *Hôpital des Enfans trouvés,* or Foundling Hospital. It is an institution for the reception of parturient females and deserted infants. Separate buildings, under the names of *Section d'Allaitement* and *Section d'Accouchement,* are respectively appropriated to these purposes. They are both under one administration; both supported by government.

About one hundred and forty beds are contained in the midwifery-department. Any woman beyond the eighth month of pregnancy, or menaced with abortion, is admitted on applying. The patients may be divided into three classes,—poor married females; the unfortunate seeking concealment; and common girls. The proportion of the latter is small. From eighteen hundred to two thousand women are annually delivered here.

"I was not a little surprized," observes Mr. Cross, "at my first entering this hospital with M. Chaussier, the chief physician, to find the wards crowded with female students. This midwifery-school was founded about twelve years ago, since which time young women have come annually from all parts of France to study there. Some pursue their education at their own expence; but most of them are chosen by the *Prefets* of the different departments, or the governors of country hospitals, by whom all expences are paid.—For six hundred *francs* these women are lodged, boarded aud educated, during one year. They reside in the hospital, and cannot go out of its precincts without permission. After a twelve-months residence, and an examination, they receive their diplomata from *l'Ecole de Medecine* to practice as midwives. Besides being practically engaged in the management of natural labours, they attend lectures given twice a week at the hospital by the Professor to *l'Ecole d'Accouchement.* They also receive instruction daily from the *Sage-femme en chef* of the hospital, and attend the course of midwifery given exclusively to them at *l'Ecole de Medecine* They follow the physician and surgeon iu their daily visits, and each *élève* makes a clinical report in writing of the patients under her care.

---

* *Dictionaire des Sciences medicales,* Article *Apothicaire.*

The accuracy and minuteness of some of these reports, presented to M. Chaussier during his visit, could not have been greater, if they had been made by an experienced practitioner. Each report contained the state of the patient at three different periods since the visit of the day before; and the state of the mind and the sensations of the patient were noticed, as well as the pulse, the skin, the bowels, the medicines administered and their effects, &c. M. Chaussier did not receive these reports as matter of form, but gave attention to the correctness of them, and made his observations upon any symptoms that appeared to have been omitted, or stated in a different way from what he expected. Besides learning the theory and practice of midwifery, the anatomy and circulation of the fœtus, and whatever is usually given in a regular course of lectures; these female students attend the dissections of *les femmes encientes ou accouchées;* who die in the hospital; and they are also instructed in the practice of phlebotomy and vaccination." Page 184—186.

Five hundred women were sent out from hence to practise in different parts of France during the first five years of the establishment. In April 1808, the hospital contained one hundred and fifty-nine students; and, in June 1814, the month of dismissal of the old and admission of the new students, one hundred and thirty, who had attended the lectures and practice of the preceding year, were examined and received tickets of qualification. At the annual examinations, prizes are adjudged to those who distinguish themselves by their attention, clinical reports, or answers to proposed questions. Many of the students, who can afford it, remain a second year at the institution; during this, they have peculiar privileges; and assist in the instruction of the younger students, who, with this view, are divided into classes. Several of these students superintend every delivery under the direction of the *sage-femme en chef.* Dubois, surgeon-accoucheur, is only summoned in extraordinary cases. No male student is ever admitted. There is an *Infermerie* for those ill during or after delivery; by the twelfth day from which the patients, under ordinary circumstances, quit the hospital.

The *Section d'Allaitement*, or Foundling Hospital, receives, besides those children whom their mothers abandon on leaving the midwifery-department, all others under two years of age, whom the parents wish to get rid of. A female porter, in constant attendance at the gate, takes in all that are brought without asking questions; and no written document is demanded. The weakly are consigned to wet nurses resident in the hospital; the robust to country nurses who take them home. Twelve thousand persons, including students, are received annually at *L'Hospice de la Maternité.* Children under two years of age, deserted by their parents, constitute one third of this number.—Any comment of ours upon the midwifery-department of this noble institution would be superfluous.

Thus far with the grand system of regular education. Over the minor and detached institutions, and places of instruction, we shall pass with a more rapid step. Four other hospitals only remain to be noticed; and we have but very little to communicate respecting them.

*L'Hôpital des Veneriens* contains six hundred beds; but is badly conducted. The care of all the patients devolves on M. Cullerier and his nephew, who reside there. They begin their visit every morning, at half past six, even in severe winter; and the men and women are separately seen. Two hundred and twenty-five of the former, *in the short space of one hour,* were examined and prescribed for by M. Cullerier; three hundred and twenty of the latter, *in less than an hour and a half,* by his nephew! Precisely sixteen seconds for each patient! This is French *volatility* with a vengeance. Happy for the poor patients if the *wings* of these fleet sons of mercury were occasionally clipped! There are separate wards for complications of itch with syphilis, and for very bad cases. The *elèves internes,* who reside in the hospital and receive salaries, accompanied the surgeons in their rounds, and noted their prescriptions. No other students were present.

*L'Hôpital des enfans malades* receives poor children, suffering from illness or accident, between infancy and the age of fifteen; such being excluded from the general hospitals. The number is generally between six and eight hundred. Both the male and female departments contain a surgical ward; a ward for acute diseases; small-pox; scrofula; tinea; and itch. The institution is admirably conducted. Clinical lectures are given during the summer; and the purchasers of tickets witness the practice of the physicians and surgeons, who visit the wards every morning, between the hours of seven and nine, during the whole year. In their absence the duties of the hospital are performed by six *elèves internes,* who reside there and receive the same salary as the other institutions. Jadelot is physician here.

*L'Hôpital St. Louis* is a large hospital in the suburbs; hence the practice of it is beneficial to few, except the resident pupils. Alibert has the charge of the wards for cutaneous diseases. He visits them three mornings in the week, about eleven o'clock; and gives a summer course of clinical lectures. His wards for diseases of the skin are constantly accessible to those only who attend his lectures.

*L'Hôpital la Salpêtrière* is an immense institution, containing between four and five thousand persons. It is destined exclusively for the reception of females. The lunatic, epileptic, febrile, aged and infirm, find an asylum within its walls. The clinical lectures, now rarely delivered there by Pinel, were once much celebrated. The epileptic wards may be visited with great advantage. *The Military Hospitals* of Paris are made subservient to the plan of instruction; and all students have access to them.

When it is considered that, in addition to these sources of knowledge, the small theatres* of *L'Hospice de Perfectionnement* are filled by such men as Magendie, Mayolin, and Beclard; that private teachers of anatomy, pharmaceutic chemistry, medical jurisprudence, and every part of medicine may be heard at small expence; that the admirable lectures of Cuvier and Blainville, on comparative anatomy, at the *Jardin des Plantes,* and those on geology, mineralogy, botany, chemistry, &c. at the same or other institutions, are gratuitously delivered; we cannot but agree with Mr. Cross, that the French capital presents opportunities of instruction in all the "branches of science which the medical student of the most elevated mind, and most liberal education, can wish to pursue."

But how have these unrivalled opportunities been improved? How this system of education, so elevated, so comprehensive, so perfect in principle, been applied to practice? Do not young men frequently elude the vigilance of the university laws, and condense the four years' study for the doctoral degree into as many weeks? What have all the boasted regulations and restrictions of the schools done for pharmacy? Do we not perpetually hear complaints, that the art is degraded and abused by a swarm of unqualified pretenders? To these queries we boldly answer in the affirmative. What too is the progress of anatomical knowledge and instruction, considered

* There are three or four of these, at which private lectures on anatomy, physiology, surgery, &c. are allowed to be delivered at hours not interfering with the regular instructions of *L'Ecole de Médecine.*

under its only important relations with the pathology of the internal or external organs? What, the actual state of practical medicine and surgery, compared with that of the healing science in this island? The former of these propositions will prove no Gordian knot to those who may take the trouble of wading through our long analysis; and they, who possess patience to accompany us to the close in our next number, will find the latter as easy of solution, convinced that in medicine, as in commerce and in arms, Britain towers high above all competition.

In some few departments of instruction and practice, however, as those of midwifery, medical jurisprudence, and comparative anatomy, France, it must be acknowledged, is decidedly before us: and fervently do we wish that, in attention to these important points, as to the general welfare of our science, the pride of British legislature and British schools would condescend to take a lesson from a rival, respectable in her inferiority, as illustrious in her misfortunes.

To enter the lists with a demonstrator upon any subject connected with anatomical instruction, may savour somewhat of presumption; but Mr. Cross has broached opinions which we deem not quite orthodox; and we are, perhaps, too little disposed to consider the character of a writer, or the perils of controversy, when error and bad doctrine demand exposure and rejection. The work of Boyer on anatomy, and the demonstrations of Beclard, are censured with some asperity by Mr. Cross: for what? why, for being too rigidly anatomical: an objection surely but little applicable to lessons of *elementary* instruction. Anatomy, physiology, and practice, he contends, should be mingled together, to make the study useful and interesting. True, Mr. Cross: but would you give compound aliment to children while their stomachs are scarcely able to digest milk? Instruction should be adapted to the intellect, as food to the system. Elementary works on anatomy, and the lessons of the demonstrator, cannot be too precise. They should possess all the rigour and correctness of botanical description. It is in these that the materials of the animal structure are to be displayed and studied in a distinct and isolated form. On the professed lecturer devolves the task of arranging such detached lessons, and combining them with physiological and pathological disquisition, so as to form one luminous and comprehensive whole: and, in like manner, the excellence of writings on medical and surgical anatomy may be estimated by the perspicuity and extent in which these complicated views of the structure, functions, and diseases, of the animal economy are developed. Each class of books, as of teachers, is directed to a distinct object; and should adopt a peculiar mode of instruction, never to be lost sight of. These are no visionary notions. In the dissecting-room they originated; in the dissecting-room they have been confirmed. And we can assure Mr. Cross, that we have known young men dissect with great ardor and advantage from the *dry* descriptions of Boyer, who turned with emptiness and disgust from the diffuse luxuriance of Bell; and gain more sound anatomical knowledge from one *dull, insipid* demonstration of the methodic Lawrence, than from half a score of the most eloquent and animated "*practical lectures*" delivered in the London schools.

---

